# Efficient replication of pneumonia virus of mice (PVM) in a mouse macrophage cell line

**DOI:** 10.1186/1743-422X-4-48

**Published:** 2007-06-04

**Authors:** Kimberly D Dyer, Ingrid MM Schellens, Cynthia A Bonville, Brittany V Martin, Joseph B Domachowske, Helene F Rosenberg

**Affiliations:** 1 Laboratory of Allergic Diseases, National Institute of Allergy and Infectious Diseases, National Institutes of Health, Bethesda, Maryland, USA; 2 Department of Pediatrics, SUNY Upstate Medical University, Syracuse, New York, USA; 3 Department of Immunology, University Medical Center Utrecht, Utrecht, The Netherlands; 4 Department of Pharmacology, University of Colorado Health Sciences Center, Denver, Colorado, USA; 5 Building 10, Room 11C215, National Institute of Allergy and Infectious Diseases, National Institutes of Health, Bethesda, Maryland 20892, USA

## Abstract

Pneumonia virus of mice (PVM; family *Paramyxoviridae*, subfamily *Pneumovirinae*) is a natural respiratory pathogen of rodent species and an important new model for the study of severe viral bronchiolitis and pneumonia. However, despite high virus titers typically detected in infected mouse lung tissue *in vivo*, cell lines used routinely for virus propagation *in vitro *are not highly susceptible to PVM infection. We have evaluated several rodent and primate cell lines for susceptibility to PVM infection, and detected highest virus titers from infection of the mouse monocyte-macrophage RAW 264.7 cell line. Additionally, virus replication in RAW 264.7 cells induces the synthesis and secretion of proinflammatory cytokines relevant to respiratory virus disease, including tumor necrosis factor-α (TNF-α), interferon-β (IFN-β), macrophage inflammatory proteins 1α and 1β (MIP-1α and MIP-1β) and the functional homolog of human IL-8, mouse macrophage inflammatory peptide-2 (MIP-2). Identification and characterization of a rodent cell line that supports the replication of PVM and induces the synthesis of disease-related proinflammatory mediators will facilitate studies of molecular mechanisms of viral pathogenesis that will complement and expand on findings from mouse model systems.

## Findings

### Background

Pneumonia virus of mice (PVM) infection in mice was originally described by Horsfall and colleagues [1,2], but until relatively recently, the sole interest in this virus was as a pathogen of laboratory rodent colonies [3-5]. Over the past several years, we and others have built on Horsfall's early studies, and have developed and characterized an *in vivo *model of severe respiratory virus infection using PVM [reviewed in [6,7]]. Among our findings, we have shown that a minimal, physiologically relevant inoculum of PVM (typically <100 pfu) results in robust virus replication in lung tissue, accompanied by influx of granulocytes in response to local production of specific proinflammatory chemokines [8]. The pathophysiology of PVM bronchiolitis leading to pneumonia and acute respiratory distress syndrome (ARDS) is similar to that observed in response to severe respiratory syncytial virus (hRSV) infection in human infants [9].

**Figure 1:**
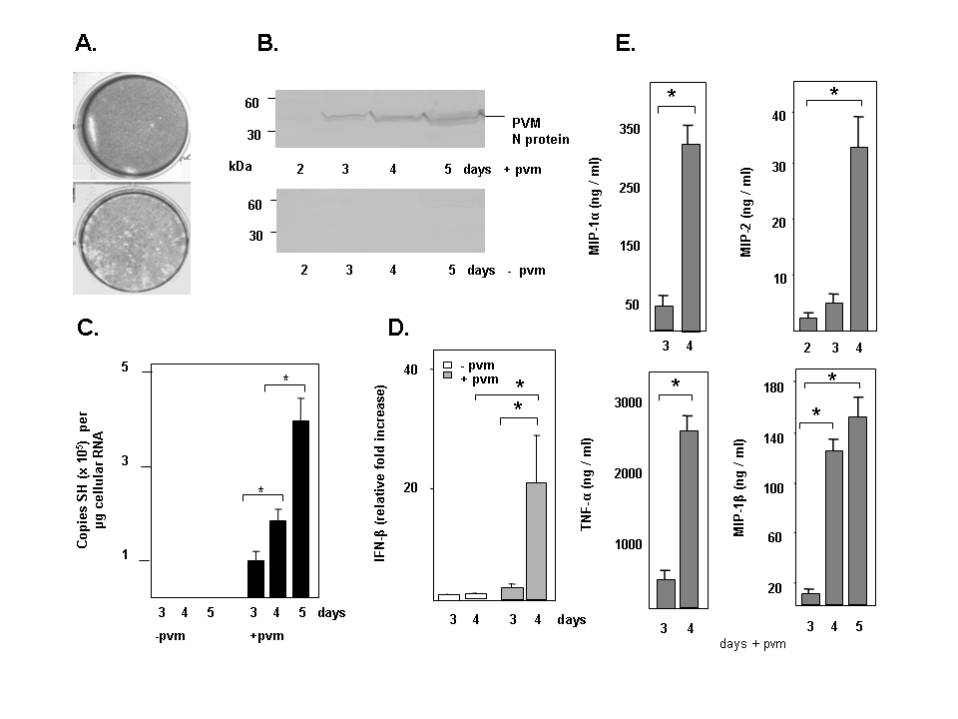
**Infection of the mouse macrophage RAW 264.7 cell line with PVM**. **(A) **A plaque assay targeting the standard BS-C-1 primate epithelial cell line (lower panel) compared to an uninfected control cell monolayer (upper panel). **(B) **Western blot of infected (+pvm) and control (-pvm) RAW 264.7 cell extracts (2 × 10^6 ^cell equivalents/lane) probed with rabbit polyclonal antisera directed against a specific 15-mer of the PVM N peptide. **(C) **Q-RT-PCR detecting PVM SH gene per microgram RNA [13] from triplicate cultures of infected (+pvm) and uninfected (-pvm) RAW 264.7 cells demonstrating ongoing PVM replication in infected cells; *p < 0.05 as indicated. **(D) **Q-RT-PCR detection of interferon-β in RNA from triplicate cultures of infected (+pvm, grey bars) and uninfected control (-pvm, white bars) cells, day 3 no infection normalized to 1.0 [13]; *p < 0.02 as indicated. **(E) **Detection of proinflammatory cytokines (MIP-1α, MIP-1β, MIP-2 and TNF-α) in culture supernatants in response to infection, data shown with background levels subtracted; *p < 0.01 as indicated.

While PVM clearly replicates efficiently in mouse lung tissue, the *in vitro *propagation of this pathogen is significantly less straightforward. The primate BS-C-1 epithelial cell line supports minimal rates of PVM replication *in vitro *[10]. The BS-C-1 cell line has been used for traditional plaque assays, but PVM-induced plaques develop slowly, have relatively indistinct borders, and are difficult to evaluate quantitatively [see Figure 1A]. Furthermore, from an evolutionary perspective, one would prefer to perform molecular studies of virus pathogenesis in cells from a relevant species, i.e...a rodent cell type or cell line. We have demonstrated that PVM replicates in the mouse LA4 respiratory epithelial cell line [11], but virus growth is similarly slow, even at temperatures permissive for virus propagation *in vitro*.

In this work, we explore PVM replication in several independent cell lines and identify the mouse macrophage RAW 264.7 cells as supporting the highest rates of virus replication. Furthermore, PVM infection of the RAW 264.7 cell line results in augmented synthesis of several pro-inflammatory mediators that are directly related to the pathogenesis of disease *in vivo*.

### The study

The rodent L2, LA4, RAW 267.4, J774A.1, RLE and 3T3 and primate A549, BS-C-1, and HEp-2 cell lines obtained from American Type Culture Collection (Manassas, VA) were maintained in Iscove's Modified Dulbecco's medium with 10% heat-inactivated fetal calf serum, 2 mM glutamine and penicillin-streptomycin at 5% CO_2 _and 32°C (permissive for virus growth in culture) unless otherwise indicated. Mouse-passaged PVM prepared as described was stored in liquid nitrogen at ~10^6 ^pfu/ml [12]. Virus replication in RAW 264.7 cells was determined by both Q-RT-PCR detection of the virus SH gene [see reference [13]] for complete method] and by western blot [14] probed with a 1:200 dilution of polyclonal anti-PVM N peptide antibody prepared against sequence SQQLNIVDDTPDDDI encoding amino acids 379 – 393 of the PVM N protein. Proinflammatory cytokines in culture were evaluated by ELISA (R&D Systems, Minneapolis, MN). Q-RT-PCR detection of interferon-β was via standard methods using primer – probe set Mm00439546_s1 (ABI, Columbia, MD) normalized as described [13] on RNA prepared from infected and control uninfected cells in culture (RNazol B, Friendship, TX).

## Results and conclusion

The cell lines evaluated for the ability to support virus replication included rat epithelial L2 and RLE, mouse epithelial LA4, mouse macrophage RAW 267.4 and J774A.1, and primate epithelial A549, BS-C-1, and HEp-2. All were inoculated with PVM on day 0 (MOI = 0.02, 10^4 ^pfu per 5 × 10^6 ^cells). On day 7, virus titer in the culture supernatants was determined by standard plaque assay [12]. Although pneumoviruses maintain strict host-pathogen specificity *in vivo*, we determined that PVM replicated to a limited extent in vitro (< 10^3 ^pfu/ml supernatant) in each of the aforementioned cell lines. The mouse monocyte/macrophage RAW 264.7 cell line (established from a tumor induced by Abelson murine leukemia virus) generated the highest virus titers (10^4 ^pfu/ml) under culture conditions described. Cells of the RAW 264.7 line also support replication of other unrelated virus pathogens, including murine hepatitis virus and Japanese encephalitis virus [15-17].

To evaluate the kinetics of virus replication and production of proinflammatory mediators in the RAW 264.7 cell line, cells at 50% confluence were inoculated with PVM (MOI 0.1) on day 0 and harvested on days 2 – 5 thereafter. RAW 264.7 is a semi-adherent cell line, and is not well-suited for plaque assays. Here, virus replication was examined qualitatively on western blot of cellular homogenates probed with PVM-specific antisera [Figure 1B]. Virus was first detected in infected cultures on day 3 post-inoculation, and then in increasing amounts through day 5. No immunoreactive PVM N protein was detected in uninfected control cultures.

Virus replication was also examined quantitatively by Q-RT-PCR using the virus SH gene as a target sequence [13], [Figure 1C]. PVM replication was readily detected in inoculated RAW 264.7 cells, reaching ~4 × 10^5 ^copies per microgram total RNA on day 5 of infection. No copies of the virus SH gene were detected in uninfected cells.

RAW 264.7 cells respond to infection with PVM by producing a variety of proinflammatory mediators. Transcription of interferon-β in response to virus infection was detected by Q-RT-PCR [Figure 1D]. Cytokines MIP-2, TNF-α, MIP-1α, and MIP-1β were detected in culture supernatants by ELISA [Figure 1E]. Interestingly, MIP-1α and MIP-2 are among the most prominent mediators detected in BAL fluid of infected mice; MIP-1α levels correlate directly with the severity of pneumovirus disease in both PVM and hRSV infection [18,19]. In parallel to our findings, hRSV replicates in the human monocytic THP-1 cell line [20], and several groups have provided evidence consistent with hRSV and bovine RSV (bRSV) replication in alveolar macrophages, although this point remains controversial [21-25]. Furthermore, hRSV infection of the human monocytic U937 cell line was associated with production of the proinflammatory mediator, platelet-activating factor (PAF) [26].

In summary, PVM has recently emerged as a useful novel model for the study respiratory disease in mice [7,27-30]; this has provided significant incentive toward identifying tissue culture systems for virus propagation. The mouse RAW 264.7 cell line supports efficient replication of PVM in vitro and responds to infection by augmenting production of cytokines implicated in the pathogenesis of respiratory disease. Use of this *ex vivo *model of PVM infection will permit further study of biological responses associated with virus infection and the cellular and molecular level.

## Abbreviations

PVM pneumonia virus of mice

IFN interferon

MIP macrophage inflammatory peptide

TNF tumor necrosis factor

RSV respiratory syncytial virus

ARDS acute respiratory distress syndrome

Q-RT-PCR quantitative reverse-transcriptase polymerase chain reaction

## Competing interests

The author(s) declare that they have no competing interests.

## Authors' contributions

KDD, IMMS, BVM and CAB performed experimental work. JBD and HFR conceived of the study, coordinated the research, and wrote and edited the manuscript. All authors read and approved the final manuscript.

## References

[B1] Horsfall FL, Ginsberg H (1951). The dependence of the pathological lesion upon the multiplication of pneumonia virus of mice (PVM). J Exp Med.

[B2] Ginsberg HS, Horsfall FL (1951). Characteristics of the multiplication cycle of pneumonia virus of mice (PVM). J Exp Med.

[B3] Boot R, van Herck H, van der Logt J (1996). Mutual viral and bacterial infections after housing rats of various breeders within an experimental unit. Laboratory Animal.

[B4] Miyata H, Kishikawa M, Kondo H, Kai C, Watanabe Y, Ohsawa K, Sato H (1995). New isolates of pneumonia virus of mice (PVM) from Japanese rat colonies and their characterization. Exp Animal.

[B5] Zenner L, Regnault JP (2000). Ten-year long monitoring of laboratory mouse and rat colonies in French facilities: a retrospective study. Lab Animal.

[B6] Easton AJ, Domachowske JB, Rosenberg HF (2004). Animal pneumoviruses: molecular genetics and pathogenesis of disease. Clin Microbiol Revs.

[B7] Rosenberg HF, Bonville CA, Easton AJ, Domachowske JB (2005). The pneumonia virus of mice (PVM) infection model for severe respiratory syncytial virus infection: identifying novel targets for therapeutic intervention. Pharmacol Therap.

[B8] Domachowske JB, Bonville CA, Gao J-L, Murphy PM, Easton AJ, Rosenberg HF (2000). The chemokine MIP-1α and its receptor CCR1 control pulmonary inflammation and anti-viral host defense in paramyxovirus infection. J Immunol.

[B9] Loughlin GM, Moscona A (2006). The cell biology of acute childhood respiratory disease: therapeutic implications. Pediatr Clin North Am.

[B10] Cook PM, Eglin RP, Easton AJ (1998). Pathogenesis of pneumovirus infections in mice: detection of pneumonia virus of mice and human respiratory syncytial virus mRNA in lungs of infected mice by in situ hybridization. J Gen Virol.

[B11] Moreau JM, Dyer KD, Bonville CA, Nitto T, Vasquez NL, Easton AJ, Domachowske JB, Rosenberg HF (2003). Diminished expression of an antiviral ribonuclease in response to pneumovirus infection *in vivo*. Antiviral Res.

[B12] Domachowske JB, Bonville CA, Gao JL, Murphy PM, Easton AJ, Rosenberg HF (2000). The chemokine macrophage-inflammatory protein-1 alpha and its receptor CCR1 control pulmonary inflammation and antiviral host defense in paramyxovirus infection. J Immunol.

[B13] Garvey TL, Dyer KD, Ellis JA, Bonville CA, Foster BS, Prussin CA, Easton AJ, Domachowske JB, Rosenberg HF (2005). Inflammatory responses to pneumovirus infection in IFNαβR gene-deleted mice. J Immunol.

[B14] Rosenberg HF, Domachowske JB, Nicholson AW (2001). Eosinophil-derived neurotoxin. Methods in Enzymology.

[B15] Pope M, Marsden PA, Cole E, Sloan S, Fung LS, Ning Q, Ding JW, Leibowitz JL, Phillips MJ, Levy GA (1998). Resistance to murine hepatitis virus strain 3 is dependent on production of nitric oxide. J Virol.

[B16] Murali-Krishna K, Ravi V, Manjunath R (1995). Japanese encephalitis virus infection of mouse cell lines: ability to prime mice for generation of virus specific cytotoxic T lymphocytes and differences in CTL recognisable viral determinants. Arch Virol.

[B17] Martin LR, Neal ZC, McBride MS, Palmenberg AC (2000). Mengovirus and encephalomyocarditis virus poly(C) tract lengths can affect virus growth in murine cell culture. J Virol.

[B18] Bonville CA, Bennett NJ, Koehnlein M, Haines DM, Ellis JA, DelVecchio AM, Rosenberg HF, Domachowske JB (2006). Respiratory dysfunction and proinflammatory chemokines in the pneumonia virus of mice (PVM) model of viral bronchiolitis. Virology.

[B19] Garofalo RP, Patti J, Hintz KA, Hill V, Ogra PL, Welliver RC (2001). Macrophage inflammatory protein-1alpha (not T helper type 2 cytokines) is associated with severe forms of respiratory syncytial virus bronchiolitis. J Infect Dis.

[B20] Krilov LR, Anderson LJ, Marcoux L, Bonagura VR, Wedgwood JF (1989). Antibody-mediated enhancement of respiratory syncytial virus infection in two monocyte/macrophage cell lines. J Infect Dis.

[B21] Panuska JR, Hertz MI, Taraf H, Villani A, Cirino NM (1992). Respiratory syncytial virus infection of alveolar macrophages in adult transplant patients. Am Rev Respir Dis.

[B22] Becker S, Soukup J, Yankaskas JR (1992). Respiratory syncytial virus infection of human primary nasal and bronchial epithelial cell cultures and bronchoalveolar macrophages. Am J Resp Cell Mol Biol.

[B23] Schrijver RS, Kramps JA, Middel WG, Langedijk JP, van Oirschot JT (1995). Bovine respiratory syncytial virus replicates minimally in bovine alveolar macrophages. Arch Virol.

[B24] Franke-Ullmann G, Pfortner C, Walter P, Steinmuller C, Lohmann-Matthes ML, Kobzik L, Freihorst J (1995). Alteration of pulmonary macrophage function by respiratory syncytial virus infection in vitro. J Immunol.

[B25] Midulla F, Villani A, Panuska JR, Dab I, Kolls JK, Merolla R, Ronchetti R (1993). Respiratory syncytial virus lung infection in infants: immunoregulatory role of infected alveolar macrophages. J Infect Dis.

[B26] Villani A, Cirino NM, Baldi E, Kester M, McFadden ER, Panuska JR (1991). Respiratory syncytial virus infection of human mononuclear phagocytes stimulates synthesis of platelet-activating factor. J Biol Chem.

[B27] Barends M, de Rond LG, Dormans J, van Oosten M, Boelen A, Neijens HJ, Osterhaus AD, Kimman TG (2004). Respiratory syncytial virus, pneumonia virus of mice, and influenza A virus differently affect respiratory allergy in mice. Clin Exp Allergy.

[B28] Claassen EA, van der Kant PA, Rychnavska ZS, van Bleek GM, Easton AJ, van der Most RG (2005). Activation and inactivation of antiviral CD8 T cell responses during murine pneumovirus infection. J Immunol.

[B29] Faisca P, Tran Anh DB, Thomas A, Desmecht D (2006). Suppression of pattern-recognition receptor TLR4 sensing does not alter lung responses to pneumovirus infection. Microbes Infect.

[B30] Bonville CA, Lao V, DeLeon JM, Gao JL, Easton AJ, Rosenberg HF, Domachowske JB (2004). Functional antagonism of chemokine receptor CCR1 reduces mortality in acute pneumovirus infection *in vivo*. J Virol.

